# Preliminary finding of a randomized, double-blind, placebo-controlled, crossover study to evaluate the safety and efficacy of 5-hydroxytryptophan on REM sleep behavior disorder in Parkinson’s disease

**DOI:** 10.1007/s11325-021-02417-w

**Published:** 2021-08-17

**Authors:** Mario Meloni, Michela Figorilli, Manolo Carta, Ludovica Tamburrino, Antonino Cannas, Fabrizio Sanna, Giovanni Defazio, Monica Puligheddu

**Affiliations:** 1 Department of Medical Sciences and Public Health, University of Cagliari, Cagliari, Italy; 2 Sleep Disorders Center, Department of Medical Sciences and Public Health, University of Cagliari, Asse Didattico E., SS 554 bivio Sestu, Monserrato, 09042 Cagliari, Italy; 3 Department of Medical Sciences and Public Health, Neurology Unit, University of Cagliari and AOU Cagliari, Monserrato, Cagliari, Italy; 4 Department of Biomedical Sciences, University of Cagliari, Cagliari, Italy

**Keywords:** Parkinson’s disease, 5-Hydroxytryptophan, REM sleep behavior disorder, Serotonin, Melatonin

## Abstract

**Purpose:**

Altered serotonergic neurotransmission may contribute to the non-motor features commonly associated with Parkinson’s disease (PD) such as sleep disorders. The 5-hydroxytryptophan (5-HTP) is the intermediate metabolite of l-tryptophan in the production of serotonin and melatonin. The purpose of this study was to compare the effects of 5-HTP to placebo on REM sleep behavior disorder (RBD) status in patients with PD.

**Methods:**

A single-center, randomized, double-blind placebo-controlled crossover trial was performed in a selected population of 18 patients with PD and RBD. The patients received a placebo and 50 mg of 5-HTP daily in a crossover design over a period of 4 weeks.

**Results:**

5-HTP produced an increase in the total percentage of stage REM sleep without a related increase of RBD episodes, as well as a marginal, non-significant reduction in both arousal index and wake after sleep onset. The self-reported RBD frequency and clinical global impression (CGI) were improved during 5-HTP and placebo treatment in comparison to baseline. 5-HTP significantly improved our patients’ motor experiences of daily living as rated by the Unified Parkinson’s Disease Rating Scale (UPDRS) part II.

**Conclusions:**

This study provides evidence that 5-HTP is safe and effective in improving sleep stability in PD, contributing to ameliorate patients’ global sleep quality. Larger studies with higher doses and longer treatment duration are needed to corroborate these preliminary findings.

**Supplementary Information:**

The online version contains supplementary material available at 10.1007/s11325-021-02417-w.

## Introduction

Sleep disturbances in Parkinson’s disease (PD) are various, multifactorial, and result in significant morbidity, including insomnia, excessive daytime sleepiness, restless legs syndrome, and REM sleep behavior disorder (RBD) [1]. The pathological loss of muscle atonia during REM sleep, namely REM sleep without atonia (RSWA), is the neurophysiological hallmark of RBD and consists of increased sustained (tonic) or intermittent (phasic) EMG activity [2]. According to current diagnostic criteria, the diagnosis of RBD requires the presence of both RSWA and dream-enactment behaviors, either documented by video-polysomnography (v-PSG) or reported by patients and/or bed partner [2].

The serotonergic system plays a crucial role in the sleep–wake cycle and its dysfunction could be responsible for the development of sleep disturbances in PD. Animal studies have demonstrated that lesions in the raphe nucleus result in reduced sleep and lower levels of serotonin [3]. Moreover, changes in raphe nuclei following sleep deprivation included increases in the neuronal size, increased firing during wake, and downregulation of 5-HT receptors [4]. Preclinical PET studies with [11C] DASB demonstrated decreased SERT binding in sleep-deprived rats [4]. Furthermore, acute RBD can be induced by the use of antidepressants, especially serotonin reuptake inhibitors (SSRI) [5], suggesting a role of the serotonergic system in the pathogenesis of RBD.

The therapeutic strategy for RBD has been limited to symptomatic treatment, mainly because the pathophysiology of RBD is far from being completely understood. Low doses of clonazepam, 0.5–1 mg at bedtime, have been considered a first-line treatment option for RBD [6]. On the contrary, a recent small-sized randomized double-blind placebo-controlled trial has not demonstrated a clear-cut efficacy of 0.5 mg of clonazepam in ameliorating probable RBD clinical status in PD patients [7].

In other open-label trials and observational studies, doses of 3–12 mg of melatonin at bedtime have been used successfully to reduce injuries, with few adverse effects [8, 9]. Conversely, a recent study did not observe an effect of 4 mg of prolonged-released melatonin for reducing clinical symptoms of RBD in PD patients [10].

The therapeutic use of 5-hydroxytryptophan (5-HTP) bypasses the conversion of l-tryptophan (LT) into 5-HTP by the enzyme tryptophan hydroxylase, which is the rate-limiting enzyme in the synthesis of serotonin. 5-HTP crosses the blood–brain barrier and it is converted to serotonin through the aromatic l-amino-acid-decarboxylase increasing central serotonin production [11].

PD is a difficult disease to treat and can cause troublesome sleep disorders in conjunction with other comorbidities such as dementia and depression [12].

Recently, we found that 5-HTP treatment improved both levodopa-induced dyskinesias and depressive symptoms in PD patients [13, 14].

However, until now, there has been inconsistent research on the use of 5-HTP in sleep-related symptoms in PD. According to these premises, we hypothesized that 5-HTP, the direct precursor of serotonin and melatonin, would have an impact on RBD, possibly improving global sleep quality and reducing both dream-enactment behaviors and RSWA, through increased serotonergic tone [15].

Here we report the results of the first double-blind and placebo-controlled crossover trial, assessing the efficacy of the 5-HTP on RBD and global sleep quality as assessed by polysomnographic nighttime sleep parameters and a specific self-administered questionnaire.

## Materials and methods

### Study design

The study protocol was conducted at the Sleep Disorders Center and Movement Disorders Center -University of Cagliari, Italy.

This is a single-center, randomized, double-blind, two-period crossover pilot study.

Treatments were identified by a number related to each random sequence. Patients, investigators, and other study members were unaware of a patient’s treatment assignment.

Safety assessments conducted throughout the study period included reports of any adverse events experienced by patients or reported by parents, together with vital signs recorded by the physician.

This study was conducted in accordance with the Declaration of Helsinki and approved by the Local Ethics Committee (PG-2016–17,064). A written informed consent was obtained from all subjects. The present study was then registered in the Eudra CT database (for details of study design, see supplementary materials).

### Participants

A total of 36 patients with PD were consecutively recruited and screened for eligibility by movement disorders specialists from the Sleep Disorders Center and Movement Disorders Center-University of Cagliari, Italy. The inclusion criteria included the presence of idiopathic PD according to the UK Brain Bank Parkinson’s Disease criteria [16] and RBD diagnosis according to the International Classification of Sleep Disorders third edition [2]. On the other hand, the exclusion criteria comprised the presence of vascular parkinsonism; brain tumor; drug-induced parkinsonism; other well-known or suspected causes of parkinsonism (e.g., metabolic) or any suggestive features of a diagnosis of atypical parkinsonism; severe dementia as defined by a MoCA (Montreal Cognitive Assessment) [17] score ≤ 18; severe speech problems and poor general health; concomitant neurologic and/or psychiatric diseases; depressed patients receiving SSRIs or SNRIs; participation in other drug studies within 30 days prior to baseline; any unstable or clinically significant condition, in the investigator’s opinion, that would impair the participants’ ability to comply with a long-term study follow-up; shift workers, who cannot ensure traditional nighttime sleep habits.

Demographic data, disease characteristics and current medication, the presence of comorbidities, and past medical history were collected during a face-to-face interview by a neurologist specialized in movement disorders, at screening and baseline evaluation. Treatment with drugs known to potentially affect RSWA, namely SSRI, SNRI, tricyclic antidepressants, benzodiazepines, melatonin, and beta-blockers, was assessed. The levodopa equivalent daily dose (LEDD) was calculated for each patient [18].

For the efficacy assessment on the overall functional status, the Unified Parkinson’s Disease Rating Scale (UPDRS, parts I, II, III, IV) scores were obtained at baseline, and at weeks 4, 8, 12, and 16 (T-end) [19]. The UPDRS part III (motor examination) was assessed 1–2 h after the patient took a scheduled dose of levodopa.

### Polysomnographic analysis

At the screening assessment, all participants underwent one full-night v-PSG in the sleep laboratory, in order to diagnose RBD. Moreover, all participants underwent a home-PSG at the end of part I (week 4) and another at the end of part II (week 12), in order to assess the efficacy of 5-HTP on RBD. In order to achieve better patient compliance and adherence to the study, we decided to perform ambulatory PSG rather than v-PSG, at week 4 and week 12. Indeed, the ambulatory PSG represents a comfortable alternative to sleep laboratory investigations.

PSG recordings, both v-PSG and home-PSG, were performed with digital polysomnography according to the American Academy of Sleep Medicine (AASM) recommendations [20] and with the same montage (for details of video-polysomnography, see supplementary materials).

### RBD clinical status assessment

All patients underwent a sleep-focused interview including RBD duration, presence of bed partner, current self-reported frequency of RBD episodes, and the clinical global impression (CGI) as a measure of RBD severity (for details of RBD clinical status assessment, see supplementary materials).

### Outcome measures

The primary efficacy outcomes were (1) the effect of 5-HTP on the percentage of RSWA compared to placebo; (2) the comparison of 5-HTP to placebo in change from baseline to weeks 4, 8, 12, and 16 in RBD clinical status, namely in self-reported frequency of RBD episodes and CGI.

The secondary efficacy outcomes were (1) the effect of 5-HTP on TST, SE, WASO, arousal index, and percentage of time in each sleep stage (N1, N2, N3, REM) compared to placebo and (2) the effect of 5-HTP on UPDRS scores.

### Statistical analysis

All subjects who completed the protocol were included in the analyses.

Given that this is a pilot study, there are no data available on 5-HTP efficacy in treating RBD in PD that allow precise sample size’s power calculations. However, based on prior studies using similar protocols [21], it is expected that a number of 18 patients, considering a ≈ 20% drop-out, with a minimum of 15 patients completing the study, would be sufficient to detect a treatment effect (paired t-test, α = 0.05, 1-β = 0.8, effect size = 0.8; G-Power 3.1).

Statistical analyses were all carried out with PRISM, GraphPad 6 Software (San Diego, USA) with the significance level set at *p* < 0.05 (for details of statistical analysis, see supplementary materials).

## Results

Out of the 36 patients with PD screened for eligibility, a total of 18 (50%) patients were diagnosed with RBD according to v-PSG baseline assessment and subsequently randomized. The mean age was 67.5 ± 7.4 years with a mean disease duration of 8.1 ± 4.7 years and a mean total LEDD of 728.6 ± 509.1. Two subjects (11.1%) were taking a low dosage of clonazepam (1 mg) and alprazolam (0.25 mg), respectively, and were maintained at the same dosage during the course of the study. No other subject was taking drugs known to potentially affect RSWA. During the study a total of 14 subjects slept with the bed partner, 3 subjects slept alone, and 1 subject did not share the same bedroom.

At the baseline evaluation, the UPDRS subsections mean scores were as follows: mean UPDRS part 1 was 2.1 ± 2.2; mean UPDRS part 2 was 7.8 ± 5.3; mean UPDRS part 3 was 16.3 ± 10.6; mean UPDRS part 4 was 3.3 ± 4.3; mean total UPDRS was 30.6 ± 18.8.

We identified the group of patients that fulfilled the criteria for the UPDRS-based tremor-dominant and akinetic-rigid subtypes of PD. Six out of 18 (33.3%) patients had an akinetic-rigid phenotype and twelve (66.6%) patients had tremor-dominant phenotype [22]. The demographic and baseline clinical information is summarized in Table 1.

**Table 1:** Baseline demographic and clinical information of the subjects randomizedto intervention (n = 18)

**Demographic and clinical information**	
Age (years)	67.5±7.4 (54-80)
Gender (M/W)	12 (66.7%) / 6 (33.3%)
PD duration (years)	8.1 ± 4.7 (2-19)
Levodopa equivalent dose (mg)	728.6±509.1 (150-1815)
Akinetic-rigid phenotype	33.3 %
Tremor-dominant phenotype	66.6 %
UPDRS I	2.1 ± 2.2 (0-8)
UPDRS II	7.8 ± 5.3 (3-25)
UPDRS III	16.3±10.6 (4-46)
UPDRS IV	3.3 ± 4.3 (0-15)
Total UPDRS	30.6 ± 18.8 (11-83)

Data are presented as mean and standard deviation (S.D) or percents

Abbreviations: *UPDRS,* Unified Parkinson’s Disease Rating Scale

Patients were randomly assigned to the placebo-5-HTP arm (n = 9) and to the 5-HTP-placebo arm (n = 9). No significant differences in baseline characteristics were detected between the 2 treatment arms.

Because of logistical issues, one subject had undergone complete withdrawal at the end of the part I period. One patient did not have the last evaluation (week 16) due to the onset of acute dehydration which required hospitalization.

**Fig. 1:**
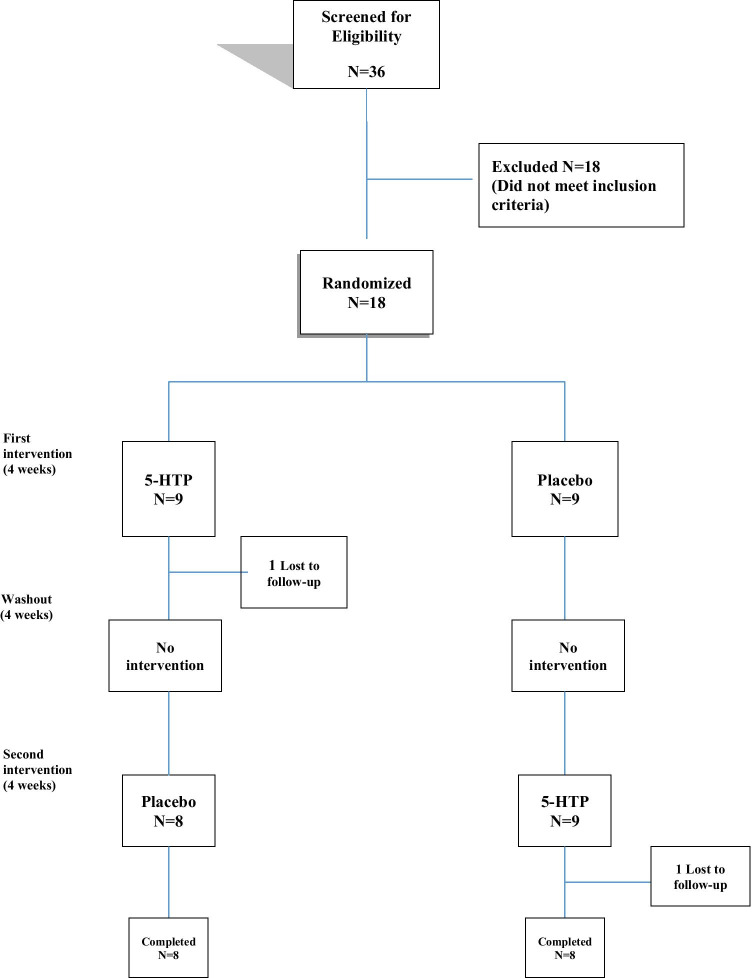
Flowchart: enrollment, randomization, and retention of study participants

Sixteen patients (11 men, 5 women) completed the study and were included in the final analysis (see Fig. 1). All 16 patients demonstrated complete adherence to the experimental treatment and remained on stable doses of dopaminergic drugs until the end of the study.

### Changes in primary outcomes

The results from the polysomnographic data and RSWA assessment are presented in Table 2.

**Table 2:** Sleep parameters and RSWA assessments

**Study outcome measures: sleep variables**				***p*** **value**		
	**Baseline**	**5-HTP**	**Placebo**	**Baseline vs 5-HTP vs Placebo**	**Baseline vs 5-HTP**	**5-HTP vs Placebo**
TST	378.1±84.3	346±76.2	323.5±85.3	.03*	*ns*	*ns*
SE	72.8±16.7	70.1±12.2	72±18.7	*ns*	*ns*	*ns*
WASO	109.8±73.7	84.1±58.3	98.9±83	*ns*	*ns*	*ns*
Arousal Index (n/h)	6.6±8.5	5.4±3.2	6.7±3.7	*ns*	*ns*	*ns*
N1 (%)	11.4±7	9.4±5	11.7±11.3	*ns*	*ns*	*ns*
N2 (%)	48.8±11.8	54.3±12.3	52±8.1	*ns*	*ns*	*ns*
N3 (%)	29.3±12.3	22.9±9.5	25±8	*ns*	.04 ^	*ns*
REM (%)	10.4±6.8	13.4±9.2	11.3±6.4	*ns*	*ns*	*ns*
REM sleep periods (n)	2.8±1.2	2.5±0.7	2.4±1.2	*ns*	*ns*	*ns*
% Any Chin	64.0±29.0	80.3±20.2	74.4±22.8	*ns*	*ns*	*ns*
%Any Chin + FDS (3 sec)	68.2±26.8	81.5±18.8	77.2±20.4	*ns*	*ns*	*ns*
% Any Chin + FDS (30 sec)	73.2±32.8	87.3±15.9	88.4±15.5	*ns*	*ns*	*ns*

Data are presented as mean and standard deviation (S.D). Abbreviations: *Any*, phasic and/or tonic; *EMG*, electromyographic; *FDS*, flexor digitorum superficialis; *TST*, Total sleep time; *SE*, Sleep efficiency; *WASO*, Wake time after sleep onset; *ns*, not significant; *Repeated measures one-way ANOVA;  ^ Paired t-test

No significant main effects of the treatment (5-HTP vs placebo) nor of time in all sleep variables evaluated have been found as reported by two-way ANOVA.

5-HTP did not significantly influence the percentage of RSWA, as assessed by the SINBAR method, compared to baseline and placebo. Indeed, 5-HTP slightly increased all RSWA parameters, although without reaching statistical significance.

Compared to baseline, 5-HTP and placebo produced a significant reduction of the CGI scores. Accordingly, one-way ANOVA revealed significant effects of treatment (F = 8.60; *p* = 0.001) and post-hoc comparisons showed a significant difference between baseline and 5-HTP treatment (t = 2.78; *p* < 0.05) as well as between baseline and placebo (t = 4.05; *p* < 0.001).

Compared to baseline, treatment with 5-HTP and placebo led to a non-significant reduction in the frequency of RBD events (disturbing dreams, dream-enactment behaviors, and somniloquy episodes) (F = 2.54; *p* = 0.09; one-way ANOVA). Results from the self-reported RBD clinical status are presented in Table 3.

**Table 3:** Self-reported RBD clinical status

**Secondary outcome measures**				***p*-value**		
	**Baseline**	**5-HTP**	**Placebo**	**Baseline vs 5-HTP vs Placebo**	**Baseline vs 5-HTP**	**Baseline vs Placebo**
**CGI**	3.23±0.59	2.76±0.83	2.53±0.87	0.001*	<0.05**	<0.001**
**Self-reported frequency**	2.66±2.05	1.60±1.18	1.53±1.06	*ns*	*ns*	*ns*

Data are presented as mean and standard deviation (S.D); *CGI*, clinical global impression; *ns*, not significant. *Repeated measures one-way ANOVA; ** post-hoc analysis

### Changes in secondary outcomes

**Fig. 2:**
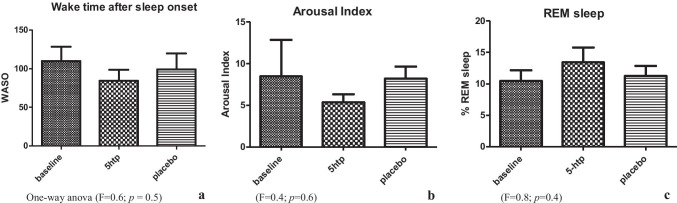
Polysomnographic sleep data: (secondaryoutcome)

The 5-HTP treatment produced an increase in the total percentage of REM sleep stage (see Fig. 2c) as well as a reduction in both arousal index and WASO compared to placebo (see Fig. 2b, 2a) without reaching statistical significance. Treatment with 5-HTP significantly (t = 2.456; *p* = 0.04) reduced the percentage of time in stage N3 compared to placebo (Table 2). There were no other significant effects of 5-HTP at the dose of 50 mg compared to placebo on nighttime sleep parameters evaluated in our study.

**Fig. 3:**
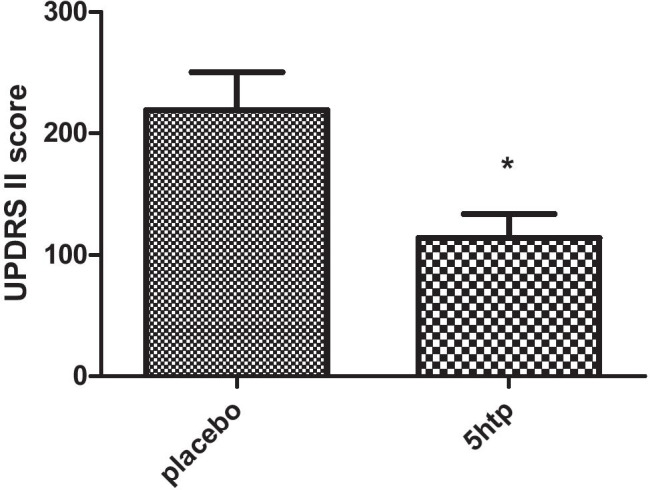
Activities of daily living rated using the UnifiedParkinson Disease Rating Scale Part II (UPDRS II) (secondary outcome)

5-HTP did not produce any significant score variations of the UPDRS part I/part III and part IV; however, it significantly improved motor experiences of daily living as rated by UPDRS part II scores, compared to placebo. Accordingly, two-way ANOVA revealed a significant time × treatment interaction (F = 4.47; *p* = 0.003), as well as significant effects of treatment (F = 8.09; *p* = 0.01) and time (F = 7.26; *p* = 0.0001), and post hoc analyses revealed significant differences between the treatment period with 5-HTP and placebo during the second part of the study (part II) (*p* < 0.01). Moreover, an unpaired t-test also detected significant differences (t = 2.83; df = 14; *p* = 0.01; see Fig. 3) when overall comparing the mean UPDRS part II scores between groups (arms) in the different treatment conditions (5-HTP vs placebo).

## Discussion

This is the first randomized, double-blind, placebo-controlled, crossover, phase II proof-of-concept clinical study investigating the effects of 5-HTP on RBD and nighttime sleep parameters in patients with PD. As reported, we have detected non-significant effects of 5-HTP on both clinical and neurophysiological RBD manifestation along with a substantial placebo effect on RBD clinical status self-reported. However, the trends of some results deserve deepening and further discussion.

In the present study, we have found a statistically non-significant increase in the total percentage of REM sleep by treatment with 50 mg 5-HTP in subjects with PD. This result is in line with previous studies in which 5-HTP has been shown to improve sleep quality by increasing REM sleep [23], which has never been replicated in PD patients with PD. Serotonin might be regarded as a positive modulator for melatonin synthesis. Indeed, the rate of pineal melatonin synthesis is dependent on the free cytoplasm pool of serotonin in pinealocytes, and the drug-induced elevation of this pool stimulates melatonin formation and increases circulating melatonin levels [15]. Actually, melatonin is synthesized from serotonin through the action of arylalkylamine N-acetyltransferase (serotonin N-acetyltransferase), and it seems that serotonin N-acetyltransferase activity is stimulated by serotonin via the 5-HT2 receptor [24].

Among all sleep stages, REM sleep is the most circadian dependent. REM sleep duration, latency, and continuity are under strong circadian control [25]. Melatonin deficit leads to instability in the circadian timing system, resulting in reduced REM sleep amount during nighttime sleep. Exogenous melatonin increases REM sleep amount in patients with reduced REM sleep duration and concurs to modulate REM sleep episode duration [26]. Moreover, the medications used in PD can also affect sleep architecture and may be associated with excessive daytime sleepiness. Indeed, levodopa can result in REM suppression and increased REM latency [27].

Furthermore, subjective reports of RBD behavioral episodes improved significantly in terms of severity, although 5-HTP has not demonstrated superiority over placebo in CGI score reduction. Self-reported frequency of RBD-related episodes, namely disturbing dreams, somniloquy, or dream-enactment motor behaviors, did not show significant reduction using 5-HTP in patients with PD, compared to baseline and placebo.

According to these scientific premises, we suggest that 5-HTP might have contributed to an increase in the total percentage of REM sleep in our patients through the increase of nocturnal melatonin levels. Coherently, despite the increase of REM sleep percentage, the number and intensity of subjective reports of RBD behavioral episodes had not increased at follow-up. In fact, melatonin was found to be effective in reducing RBD episodes [8, 21].

In the present study, we have also found non-significant changes in RSWA parameters by 5-HTP in patients with PD, compared to baseline and placebo. The exact pathogenesis of RSWA is not yet completely understood. The physiological REM sleep atonia is thought to be triggered by glutamatergic and GABAergic/glycinergic brainstem inputs, by means of both motoneuron inhibition and reduced motoneuron excitation [28]. A decrease of serotoninergic activity is thought to be implicated in REM atonia mechanisms [28] and melatonin may reduce the RSWA in RBD [21].

We hypothesized that our findings on RSWA might be explained by the dual and counterbalanced increase of serotonin and melatonin in the central nervous system. Indeed, the increment of serotonergic tone may increase motoneuron activity during REM sleep-inducing RSWA [28].

In our study, a non-significant trend for decreased arousal index and the amount of WASO was found in subjects who received 5-HTP compared to placebo, contributing to ameliorate the global sleep quality. In fact, 5-HTP could improve sleep quality by modulating the arousal level and by reducing sleep instability through stabilization of sleep microstructure [29]. It is tempting to speculate that the global sleep quality improvement could explain the significant improvement in activities of daily living as assessed by UPDRS part II and the significant amelioration of depressive symptoms as reported in a parallel clinical study [13].

Moreover, patients receiving 5-HTP compared to baseline have shown a significantly reduced amount of NREM sleep stage N3 compare to baseline. The latter result might be associated with the effect of 5-HTP in modulating the frequency band and the spectra of delta waves [29]. Indeed, 5-HTP has been shown to be effective in the management of NREM sleep parasomnia, which is related to delta sleep instability [30].

The low dosage (50 mg/day) of 5-HTP may represent a limitation. In fact, the interesting trends to improve several clinical outcomes that we found may suggest that the dose of 5-HTP was too low to induce a complete beneficial effect.

Another possible limitation of this study is that all patients underwent one full-night attended v-PSG recording in the sleep laboratory with digital polysomnography, while the subsequent PSG recordings were home based. This workup setting may have caused heterogeneity of results due to a different sleep environment. However, it has been reported that RSWA has a good night-to-night agreement and seems to be more stable than video-recorded behavioral manifestations across nights [31]. Furthermore, in order to assess treatment efficacy, we analyzed the self-reported frequency of RBD episodes and the clinical global impression. A validated scale exploring RBD severity in terms of frequency and intensity of episodes is particularly needed in both clinical practice and the research field.

A further limitation of our study is the small size of our sample.

Finally, the exact dosage and duration of 5-HTP supplementation in the PD-RBD population has yet to be established.

In conclusion, our data show that 5-HTP supplementation may be effective in reducing subjective impression of the severity and frequency of the RBD episodes in patients with PD and RBD.

Nonetheless, further studies are required to confirm these preliminary data.

## Supplementary Information

Below is the link to the electronic supplementary material.

ESM 1 (DOCX 23 kb)

## Data Availability

The data that support the findings of this study are available from the corresponding author upon reasonable request.
