# Supplementary material for: Preliminary finding of a randomized, double-blind, placebo-controlled, crossover study to evaluate the safety and efficacy of 5-hydroxytryptophan on REM sleep behavior disorder in Parkinson’s disease
Source: Sleep Breath. 2021 Aug 17;26(3):1023–31. doi: 10.1007/s11325-021-02417-w (PMC9418091; doi:10.1007/s11325-021-02417-w)
Supplement: Supplementary file 1 — (DOCX 23 kb) [file 11325_2021_2417_MOESM1_ESM.docx]

**Study design:**

As schematically illustrated in Fig. 1, the study included six visits for each patient. At screening (visit one), all participants underwent a full-night attended video-polysomnographic (v-PSG) recording at a sleep laboratory and a comprehensive clinical assessment in order to verify inclusion and exclusion criteria. At the second visit (baseline), the patient who meets the eligibility criteria was randomized on day 1 in a 1:1 ratio to receive a placebo or 5-HTP and assigned a randomization study number. At this time, the patients initiated their first treatment period, receiving either 5-HTP or placebo for 4 weeks (we referred to this part of the study as Part I), according to their randomization, until their third visit. After a washout period of 4 weeks, the patients crossed over to receive the alternative treatment for a further 4 weeks, placebo capsule (matching 5-HTP) or 5-HTP 50 mg capsule, respectively, once daily (we referred to this second part of the study as Part II). The washout period was 4 weeks. Indeed, the half-life of 5-HTP is relatively short (4.3 ± 2.8 hours) [1], and its time to maximal concentration is 1-2 hours [2].

Patients were assessed at screening, baseline (week 0), weeks 4, 8, 12 (end of treatment) and 16 (T-end). Throughout the entire study, our patients were asked not to change their antiparkinson drugs and daily routines. The treatment was administered orally once a day at night, 30 minutes before bedtime and approximately 45 to 60 minutes after the last daily dose of levodopa.

The 5-HTP and placebo were dispensed in identical sealed and coded packages, inserted in indistinguishable capsules in terms of appearance, smell and taste. The capsules were manufactured and analyzed by the hospital affiliated pharmacy according to the randomization schedule and were then supplied to the trial clinicians. Hard capsules of animal gelatin were used and a mixture of excipients not containing lactose was chosen as follows: pregelatinized corn starch, calcium carbonate, magnesium stearate and silica.

**VPSG scoring details:**

The v-PSG was performed with digitally synchronized videography. For all recordings, the following montage was used: electroencephalographic leads (F3-A2, F4-A1, C3-A2, C4-A1, O1-A2, O2-A1), left and right electrooculography (EOG) channels, bilateral surface EMG channels (submentalis, flexor digitorum superficialis on upper limbs, tibialis anterior on lower limbs), and electrocardiography. The respiratory analysis included nasal airflow, which was recorded by both thermistor and nasal pressure sensor, thoracic and abdominal respiratory effort, oxygen saturation recording by cutaneous finger pulse-oximeter and microphone. In order to improve the detection of motor activity during v-PSG, patients slept uncovered. Furthermore, we allowed a light sheet for their comfort.

RSWA was quantified in patients having spent at least 5 minutes in REM sleep since shorter REM sleep duration was believed to be inadequate for a consistent assessment of RSWA.

RSWA was defined by the presence of ≥27% of 30-s REM sleep epochs containing, “any” (either tonic or phasic) chin EMG activity, combined with bilateral phasic activity of the flexor digitorum superficialis (FSD) muscle, according to the SINBAR scoring method [3] that was suggested by the ICSD-3 “as the most current evidence-based data for detecting RSWA in the evaluation of RBD, reliably distinguishing RBD patients from controls”. Moreover, according to the SINBAR method, RSWA is also defined by either ≥18% of 3-s REM sleep mini-epochs with any chin EMG activity and by ≥ 32% of 3-s REM sleep epochs containing any chin EMG activity combined with bilateral FDS EMG activity.

Each video-recorded REM sleep period was carefully visually analyzed in order to detect any motor behaviors or sleep vocalizations referable to RBD, such as violent and non-violent motor complex activity. REM sleep epochs were carefully examined for artifacts, like increases in EMG tone caused by respiratory arousal. The minimum amplitude of EMG activity during NREM sleep was considered as the background EMG activity for each patient. The EMG signal was analyzed with a notch filter at 50 Hz and rectified.

Sleep stages were scored according to AASM criteria with allowance to chin EMG muscle tone during REM sleep. The following sleep data were collected for descriptive purpose: total sleep time (TST), sleep efficiency (SE), wake after sleep onset (WASO), percentage of time in each sleep stage (N1, N2, N3, REM), number of REM sleep episode and arousal index.

RBD diagnosis was made according to the International Classification of Sleep Disorders third edition (ICSD-3) including quantification of RSWA [4]. RSWA was manually quantified according to the SINBAR scoring method [5]. Each video-recorded REM sleep period was carefully visually analyzed in order to detect any motor behaviors or sleep vocalizations referable to RBD.

RBD diagnosis, sleep scoring and RSWA assessment were performed by neurologist specialized in Sleep Medicine (MF) who was blinded to clinical patient's status.

**RBD Clinical status assessment**

A sleep-focused interview including RBD duration, presence of bedpartner, current self-reported frequency of RBD episodes and the CGI were administered.

The self-reported frequency was assessed using a 4-weeks log in which the patient was required to describe every morning any disturbing dream, somniloquy or dream-enactment motor behaviors occurred the night before. In particular, self-reported frequency was scored as follow: 0 if the patient reported no dream, any somniloquy or any dream-enactment motor behaviors; 1 if less than once RBD events (disturbing dream, somniloquy and dream-enactment motor behaviors) per week over each 4 week study; 2 if 1-3 RBD events per week, 3 if 3-7 RBD events per week or 4 if more than 7 RBD events per week. CGI was measured as follows: 1=no RBD symptoms, 2=minimal/nearly absent, 3=mild; 4=moderate; 5=marked, 6=severe, 7= very severe RBD clinical perception.

RBD clinical assessment was done at baseline, 4, 8, 12 weeks and 1 month after the end of the study (week 16).

**Statistical Analysis**

To evaluate the effect of 5-HTP and placebo on all of the primary and secondary outcome measures, two-way ANOVAs for repeated measures, with time as the within-subjects factor and treatment (5-HTP or placebo) as the between-subjects factor, were at first performed.

Moreover, follow-up one-way ANOVAs for repeated measures were performed in order to evaluate the differences between all 5-HTP (Part 1 and Part 2) and placebo (Part 1 and Part 2) treatment conditions compared to baseline data from both groups or the scores in the different treatment conditions (5 HTP vs placebo) for each experimental group (5-HTP-placebo and placebo-5-HTP).

Post-hoc comparisons were performed by means of Bonferroni’s corrected t-tests, according to the significance of main factors and/or interactions. When appropriate, additional t-tests on selected paired comparisons were also performed.

**References**

1. Westenberg HGM, Gerritsen TW, Meijer BA, van Praag HM (1982) Kinetics of l-5-hydroxytryptophan in healthy subjects. Psychiatry Research. https://doi.org/10.1016/0165-1781(82)90074-9

2. Magnussen I, Van Woert MH (1982) Human pharmacokinetics of long term 5-hydroxytryptophan combined with decarboxylase inhibitors. European Journal of Clinical Pharmacology. https://doi.org/10.1007/BF01061381

3. Frauscher B, Ehrmann L, Högl B (2013) Defining muscle activities for assessment of rapid eye movement sleep behavior disorder: From a qualitative to a quantitative diagnostic level. Sleep Medicine. https://doi.org/10.1016/j.sleep.2012.09.028

4. Medicine AA of S (2014) International Classification of Sleep Disorders 3nd edition. Diagnostic and coding manual.

5. Frauscher B, Iranzo A, Gaig C, et al (2012) Normative EMG values during REM sleep for the diagnosis of REM sleep behavior disorder. Sleep 35:835–847. https://doi.org/10.5665/sleep.1886
